# Mediator Effects of Cognitive Load on Association between Self-Efficacy and Task Load in Intensive Care Unit Nurses

**DOI:** 10.1155/2024/5562751

**Published:** 2024-02-02

**Authors:** P. Tingting, D. Xun, W. Jun, D. Shu, Z. Shan

**Affiliations:** ^1^Cardiac Center, Beijing Anzhen Hospital, Beijing 100029, China; ^2^Respiratory Intensive Care Unit, Xuan Wu Hospital, Beijing 100053, China; ^3^Department of Cardiology, Beijing Chao-Yang Hospital, Beijing 100020, China; ^4^School of Nursing, Capital Medical University, Beijing 100069, China

## Abstract

**Aims:**

To explore the mediating effect of cognitive load on the relationship between self-efficacy and task load among intensive care unit (ICU) nurses.

**Background:**

Studies related to ICU nurses' self-efficacy, cognitive load, and task load are noteworthy but limited.

**Methods:**

A total of 253 ICU nurses from three tertiary hospitals in Beijing were recruited and investigated by the Chinese version of the National Aeronautics and Space Administration-Task Load Index (NASA-TLX), General Self-Efficacy Scale (GSES), and an instrument for Measuring Different Types of Cognitive Load (MDT-CL) scales. SPSS 25.0 was used for Pearson correlation analysis and multiple linear regression analysis and mediation effect analysis using Model 4 in PROCESS (5,000 resamples).

**Results:**

Mediation analysis indicated that a partial mediating effect of extraneous cognitive load between self-efficacy and task load among ICU nurses was −0.707 (95% CI: −0.940, −0.504), accounting for 51.64% of the total effect.

**Conclusion:**

This study suggests that enhancing ICU nurses' self-efficacy can be a potential strategy to decrease extraneous cognitive load and task load. *Implications for Nursing Management*. Nursing administrators should actively implement intervention strategies based on influencing the task load pathway of ICU nurses to ensure they can provide safe and high-quality nursing services.

## 1. Introduction

The demand for nursing services is constantly increasing, and nurses are the most direct personnel to provide nursing care to patients, especially in intensive care units (ICUs) where nursing activities are characterized by extremely demanding workloads [1, 2]. ICU nurses need to possess the ability to make clinical decisions quickly, handle complex changes in patient conditions, and use special equipment. The complexity and urgency of the ICU environment often expose nurses to high-level mental task loads [1, 3]. Paula et al. [3] investigated 111 ICU nurses and 100% indicated a medium to high level of mental task load. Nursing administrators and researchers are paying more attention to the task load of nursing care. High-level task loads among ICU nurses can lead to physical and psychological problems, such as the increased risk of anxiety and depression, higher occupational fatigue and turnover rate, missed nursing care, and increased nursing errors [4, 5]. For example, Boehm and colleagues surveyed 268 ICU nurses and found that for every additional point increase in task load (rated on a scale of 0–10), adherence to bundle interventions among ICU nurses decreased by 53% [4].

Studies have shown that self-efficacy and cognitive load were related to an individual's mental task load [6–8]. Self-efficacy is defined as the level of belief in one's ability to complete a heavy workload successfully [9]. Cognitive load is defined as the total amount of cognitive resources that a person requires to process cognitive tasks [10] and is composed of intrinsic, extraneous, and germane cognitive load [11]. Intrinsic cognitive load is defined as the demand for working memory in processing information elements and their interactions in related tasks [12]. Extraneous cognitive load is generated by inappropriate activity presentation rather than the task itself [12]. Germane cognitive load increases schema construction, therefore promoting the execution of the task [12, 13]. Broad evidence has shown that self-efficacy also affects an individual's cognitive load [14, 15]. Jiang et al. [14] reported that self-efficacy in English reading among students is negatively related to intrinsic cognitive load. However, the mediating effect of cognitive load between self-efficacy and task load is unclear. Therefore, the study aimed to investigate the mediator effects of cognitive load on the association between self-efficacy and task load of ICU nurses.

## 2. Materials and Methods

### 2.1. Study Design and Samples

A cross-sectional questionnaire study design was conducted with convenience sampling at three tertiary hospitals between February and June 2023.

Registered nurses were eligible for the study if they consented to participate in this study. Nurses were excluded if they (a) had less than 1 year of intensive care experience, (b) currently did not work full-time in the ICU, and (c) had long leave with pay, such as sick or prenatal leave. A simple structural equation model (SEM) was used to analyze the relationship among self-efficacy, cognitive load, and task load, and a minimum of 200 patients was required to run the model as suggested by Shah and Goldstein [16].

### 2.2. Instruments

The questionnaire consisted of four parts: (a) demographic information; (b) the Chinese National Aeronautics and Space Administration-Task Load Index (NASA-TLX); (c) the Chinese General Self-Efficacy Scale (GSES); and (d) an instrument for Measuring Different Types of Cognitive Load (MDT-CL). Cronbach's *α* coefficient for the combined questionnaires was 0.732, indicating that the Chinese version of the NASA-TLX, GSES, and MDT-CL had acceptable reliability for ICU nurses.

### 2.3. Demographic Information

Demographic information included age, gender, education level, marital status, number of children, living situation, years of ICU working experience, professional title, number of night shifts per month, and number of patients cared for during the whole shift.

### 2.4. National Aeronautics and Space Administration-Task Load Index

The NASA-TLX was used to assess task load and was developed by Hart [17] and was translated into Chinese by Liling Liang and colleagues [18]. The NASA-TLX has 6 items: mental demand, physical demand, temporal demand, performance, effort, and frustration. Each item is scored on a Likert scale from 0 (low load) to 20 (high load), and the total score ranges from 0 to 120, with higher scores indicating a higher level of task load. The retest reliability of the scale was 0.806, and Cronbach's *α* coefficient was 0.707 [18]. Content validity in the Chinese version of NASA-TLX was 0.900, and the split-half reliability was 0.808 [19]. The Chinese version of NASA-TLX has good consistency between each item and validity.

### 2.5. General Self-Efficacy Scale

The GSES was developed by Schwarzer [9] and was translated into Chinese version by Caikang Wang et al. [20]. The GSES consists of 10 items, and each item is scored on a 4-point Likert scale ranging from 1 (“not at all true”) to 4 (“exactly true”). Example items are “If you try hard, you can always accomplish a task efficiently” and “Even objected by others, you still can manage to get what you want.” A single score for the GSES is computed using the total score of all items. A higher total score indicates a higher level of self-efficacy. The Chinese version of the GSES has shown good reliability and validity for Chinese adults [20, 21]. Cronbach's *α* coefficient was 0.870, retest reliability was 0.830 (*P* < 0.001), and split-half reliability was 0.820 (*n* = 401, *P* < 0.001) [20].

### 2.6. An Instrument for Measuring Different Types of Cognitive Load

The MDT-CL was used to assess three types of cognitive load. The MDT-CL was developed by Leppink and colleagues [12] and was translated into Chinese version by Zhang et al. [13]. A ten-item questionnaire was presented for the measurement of intrinsic cognitive load (items 1, 2, and 3), extraneous cognitive load (items 4, 5, and 6), and germane cognitive load (items 7, 8, 9, and 10). Example items are “The intensive care activity/activities presented in daily usual care was/were very complex” for intrinsic cognitive load, “The instructions and/or explanations in daily usual care during implementing intensive care activity/activities were very unclear” for extraneous cognitive load, and “Daily usual care enhanced my understanding of the intensive care activity/activities covered” for germane cognitive load. Each item scores from 0 (“not at all”) to 10 (“completely”), the higher the score, the higher the cognitive load. The Chinese version MDT-CL has good reliability and validity, and Cronbach's *α* was 0.818; Cronbach's *α* in measurement of intrinsic, extraneous, and germane cognitive load was 0.879, 0.878, and 0.946, respectively [13].

### 2.7. Data Collection

Before the study commencement, the investigators received research training about how to use standardized instruments to ensure the collected data were consistent and accurate. Data collectors recruited ICU nurses based on the inclusion and exclusion criteria, and the aim and procedure of the study were fully explained to all eligible nurses before the recruitment. This survey was conducted anonymously, utilizing online responses via mobile phones. To ensure the accuracy and effectiveness of the questionnaire, any questions related to the study were answered by the investigators during the completion of the questionnaire. All of the questions refer to the daily nursing activity that just finished. For instance, “The nursing activity presented in daily usual care that I perceived as very complex,” “The instructions and/or explanations in daily usual care were, in terms of clinical application, very ineffective,” and “Daily usual care enhanced my knowledge and understanding of nursing.” After completing the questionnaire, all data were entered into a database by two researchers who conducted extensive error and validity checks. The quality of the questionnaires was checked, and any questionnaires with identical answers or missing answers to more than two-thirds of the questions were excluded.

### 2.8. Informed Consent and Ethical Considerations

The institutional review board of the university approved this study (approval number: Z2019SY021). Written informed consent was obtained from all ICU nurses after the investigators introduced the study procedures in detail. The eligible ICU nurses had the right to withdraw from the study at any time without any harmful consequences.

### 2.9. Data Analysis

The SPSS version 25.0 (SPSS Inc., Chicago, IL) was used for data analyses, cases with missing data in self-efficacy, cognitive load, and task load, or with missing covariates (age, gender, educational level, and years of ICU working experience) >35% were excluded from the final analysis, and others were processed using multiple imputations if the missing is in random [22]. Continuous variables were described as means and standard deviation (SD) for normal and medians and interquartile range for abnormally distributed data. The relationship among self-efficacy, cognitive load (intrinsic, extraneous, and germane cognitive load), and task load (dichotomous variable) was verified using the Pearson correlation test. Finally, the significant factors (*P* < 0.05) were selected to enter a multiple linear regression analysis. The standardization coefficient (*β*) and standard error (SE) were used to express the strength of the association. Finally, all significant variables that were identified by the multivariate linear regression were entered into the structural equation model (SEM). Harman's single-factor test was used to examine common method variance with self-report measures [23]. Model 4 test in PROCESS in SPSS (an add-on for SPSS) was used for mediation analysis of task load, self-efficacy, and cognitive load [24]. The bootstrap method with a 95% bias-corrected confidence interval (CI) was used to test the significance of the mediation effect. The statistical significance was set at *P* < 0.05, with two-tailed testing.

## 3. Results

### 3.1. General Characteristics of the Participants

A total of 265 questionnaires were distributed, and 253 (95.47%) valid questionnaires were returned. The average age was 32.91 years (SD = 6.30; range between 21 and 52 years), and 65.3% are female (Table 1). The mean years of ICU working experience were 9.81 years (SD = 5.18), and most of the ICU nurses (86.6%) reported working night shifts.

### 3.2. Correlation among Major Variables

The ICU nurses showed high levels of task load with an overall NASA-TLX score of 86.49 ± 15.05, and 42.69% (108/253) of ICU nurses had a high-level task load (overall NASA-TLX score ≥90). The score of each item was as follows: mental demand (15.17 ± 3.30), physical demand (16.87 ± 2.97), temporal demand (15.46 ± 3.32), performance (16.01 ± 2.92), effort (11.47 ± 5.96), and frustration (11.49 ± 5.47).

As shown in Table 2, statistically significant differences were observed between task load and self-efficacy (*r* = −0.437, *P* < 0.001), task load and intrinsic cognitive load (*r* = 0.373, *P* < 0.001), and task load and extraneous cognitive load (*r* = 0.636, *P* < 0.001). However, no significant differences were found between task load and germane cognitive load (*r* = −0.051, *P* > 0.05).

### 3.3. Factors Affecting Task Load

The self-efficacy, intrinsic cognitive load, and extraneous cognitive load were entered into regression analysis with enter forwards selection due to these factors being statistically related to task load. As shown in Table 3, after adjusting covariates (age, gender, educational level, and years of ICU working experience), the results indicated that only self-efficacy (*β*′ = −0.202, *P* < 0.001) and extraneous cognitive load (*β*′ = −0.520, *P* < 0.001) were statistically explained task load.

### 3.4. Structural Model

Harman's single-factor test showed that the cumulative variance interpretation was 28.26%, which is lower than the critical standard of 40% [25], illustrating that common method variance was not significant in this study.

**Figure 1:**
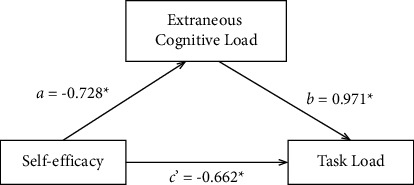
Mediation model of extraneous cognitive load on the relationship between self-efficacy and task load of ICU nurses. Note: All coefficients are significant (*P* < 0.001).

Figure 1 shows the mediation model.

The direct and indirect effects of the model are summarized in Table 4. The results showed that self-efficacy significantly predicted extraneous cognitive load (*a* = −0.728, SE = 0.102, *P* < 0.001), extraneous cognitive load was shown to be a significant predictor of task load (*b* = 0.971, SE = 0.093, *P* < 0.001), and self-efficacy also had a direct effect on task load (*c*′ = −0.662, SE = 0.163, *P* < 0.001). The bias-corrected percentile Bootstrap method test showed that extraneous cognitive load partially mediated the relationship between self-efficacy and task load (*a∗b* = −0.707, 95% CI: −0.940, −0.504; SE = 0.112; *P* < 0.001). The mediation effect accounted for 51.64% of the total effect.

## 4. Discussion

In this cross-sectional design study, 253 ICU nurses were investigated to explore the mediator effects of cognitive load between self-efficacy and task load. We found that 42.69% of ICU nurses had high levels task load, and correlations analysis reported self-efficacy was negatively related to task load, but intrinsic and extraneous cognitive load were positively related to task load. However, after adjusting covariates in multiple linear regression analysis, only self-efficacy and extraneous cognitive load were statistically explained task load. A three-factor mediation model was constructed and tested, and the relationships between self-efficacy and task load were partially mediated by extraneous cognitive load among ICU nurses, accounting for 51.64% of the total effect. The findings imply that even though the perceived task load of ICU nurses was affected by both poor self-efficacy and high-level extraneous cognitive load from daily intensive care activities, the influence of self-efficacy can be increased if extraneous cognitive load declines. Therefore, early assessment of self-efficacy and cognitive load for ICU nurses may benefit from targeted prevention strategies.

The partially mediating role of extraneous cognitive load between overall self-efficacy and task load of ICU nurses was confirmed in our study. In other words, an individual's self-efficacy not only has a direct effect on task load but also has an indirect effect through extraneous cognitive load. ICU nurses with high-level self-efficacy are always able to cope with unexpected issues and come up with solving strategies [26, 27]. These abilities weaken the high level of the extraneous cognitive load caused by unclear instructions for nursing activities in daily nursing practice, thereby reducing the task load in terms of mental demand, physical demand, temporal demand, performance, effort, and frustration for ICU nurses. Highlighting the meaningfulness of high-level self-efficacy and low-level extraneous cognitive load might contribute to a decreased perceived task load.

Regarding the relationship between self-efficacy and perceived task load among ICU nurses, it has been found that high levels of self-efficacy were associated with lower levels of task load. The findings from this study were confirmed by other studies [7, 8]. Cayupe et al. [7] recruited 300 primary school teachers to examine the mediating role of job satisfaction between self-efficacy, life satisfaction, workload, and overall life satisfaction. They also found that self-efficacy was negatively related to workload (*r* = −0.43). In addition, Molero et al. [8] investigated 1307 nurses in a cross-sectional study and reported that workload had a significant negative relationship with perceived self-efficacy (*r* = −0.07; *P* < 0.01).

It is likely because ICU nurses with high-level of self-efficacy are more capable of coping with the workload brought on by the high-intensity working environment. They are more likely to adopt proactive strategies to alleviate challenges from intensive tasks, such as participating in education programs, flexibly adjusting resource allocation, generating effective work plans, and time management strategies, and having good team cooperation ability; these strategies could help ICU nurses alleviated the perceived task load [28–30]. Therefore, the nursing administrator should provide job support to increase self-efficacy among ICU nurses, which would empower and motivate them to cope with an intensive task load.

Our study also examined the correlation between extraneous cognitive load and task load, indicating that the high level of extraneous cognitive load from unclear instructions in daily intensive care activities was positively related to higher perceived task load. Nasirizad and colleagues [31] conducted a cross-sectional study among 105 ICU nurses. They confirmed a significant relationship between cognitive and task load (*P* < 0.001). Based on the Cognitive Load Theory (CLT), the human cognitive resources are limited, and once the cognitive resources required for the cognitive tasks to be processed exceed the total cognitive resources of human beings, cognitive load increases and results in unsatisfactory task performance [10]. The extraneous cognitive load can be derived from suboptimal presentation methods and unclear instruction of activities [12]. Therefore, nursing administrators should identify and modify the factors that increase the extraneous cognitive load in nursing practice, for example, by simplifying nursing processes and improving collaboration mechanisms. In addition, administrators can introduce appropriate information technology-assisted tools, such as intelligent devices, to improve the presentation mode of interventions, provide decision support, and alleviate the nurse's extraneous cognitive load [32, 33].

### 4.1. Limitations and Future Research

This study has several limitations. First, the ICU nurses were recruited from three tertiary hospitals in one region, which limits the generalisability of the study findings to different populations and regions. In the future, expanding the sample collection is recommended to identify differences between region levels. Second, the nature of the cross-sectional study limited the possibility of drawing causal relationships among the variables. To gain a better understanding of the relationship between self-efficacy, cognitive load, and task load in ICU nurses, prospective longitudinal studies were recommended to confirm causal relationships in the future. Finally, the investigated variables in this study were collected by self-report questionnaires, which may lead to recall bias. For future studies, objective measurements of task load, such as pupil diameter, heart rate variability (HRV), and electroencephalography (EEG), are recommended [34, 35].

## 5. Conclusions

In this cross-sectional study, we concluded that self-efficacy and lower extraneous cognitive load could help defend against the adverse impact of increased task load among ICU nurses. Self-efficacy influences the task load, and extraneous cognitive load partially mediates the correlation. Therefore, nursing managers should strengthen the assessment and monitoring of self-efficacy and cognitive load in ICU nurses and tailor effective support strategies to reduce task load. To sum up, self-efficacy is a crucial resource in the ICU nursing work environment with positive impacts on well-being (e.g., reducing physical demand and frustration).

## 6. Implications for Nursing Management

Given that self-efficacy has beneficial consequences for nurses' cognitive load and task load, interventions directed at three factors can be carried out. Nursing administrators need to pay attention to the measurement of nurses' self-efficacy, cognitive load, and task load, focus on the presentation of nursing activities and other factors that may affect the source of task load for ICU nurses, and provide targeted intervention measures. For example, nursing administrators should promote the development of strong cooperation among nursing teams (e.g., gratitude for the help and mutual appreciation) to work together in high-pressure and complex ICU work environments. These effective strategies for enhancing self-efficacy and improving the cognitive load of nurses can help them to reduce work burden and pressure, thereby improving their job satisfaction, physical and mental health, and work performance.

## Figures and Tables

**Table 1:** The sociodemographic characteristics of ICU nurses (*N* = 253).

Variables	*n* (%)
*Gender*	
Male	88 (34.7)
Female	165 (65.3)

*Age (years)*	
20–30	77 (30.4)
31–40	152 (60.1)
>40	24 (9.5)

*Ethnicity*	
Han	239 (94.6)
Manchu	7 (2.7)
Others	7 (2.7)

*Education level*	
High school	74 (29.2)
Bachelor degree	179 (70.8)

*Marital status*	
Married	162 (64.0)
Unmarried	84 (33.3)
Divorce	7 (2.7)

*ICU working experience (years)*	
1–5	51 (20.2)
6–10	91 (36.0)
11–15	74 (29.2)
>15	37 (14.6)

*Living situation*	
Living with family	206 (81.4)
Alone	13 (5.2)
Share-house with others	34 (13.4)

*Working night shifts*	
Yes	219 (86.6)
No	34 (13.4)

*Number of night shifts per month*	
0	27 (10.7)
1–5	7 (2.7)
6–10	209 (82.6)
≥11	10 (4.0)

*Number of patients cared for during the day shift*	
0–2	239 (94.5)
≥3	14 (5.5)

*Number of patients cared for during the night shift*	
0–2	250 (98.8)
≥3	3 (1.2)

*Professional title*	
Primary nurse aide	37 (14.6)
Senior nurse	101 (39.9)
Supervisor nurse	115 (45.5)

*Average overtime hours per week*	
0	30 (11.8)
1–5	81 (32.0)
5–10	54 (21.3)
10–15	67 (26.5)
>15	21 (8.3)

*ICU specialty nurse*	
Yes	104 (41.1)
No	209 (58.9)

**Table 2:** Descriptive statistics and correlations of the study variables (*N* = 253).

	1	2	3	4	5
(1) Self-efficacy	1.000				
(2) Intrinsic cognitive load	−0.332^*∗∗*^	1.000			
(3) Extraneous cognitive load	−0.410^*∗∗*^	0.474^*∗∗*^	1.000		
(4) Germane cognitive load	0.221^*∗∗*^	0.053	−0.262^*∗∗*^	1.000	
(5) Task load	−0.437^*∗∗*^	0.373^*∗∗*^	0.636^*∗∗*^	−0.051	1.000
Mean	25.166	23.593	16.233	34.968	86.458
Standard deviation	4.805	7.444	8.495	7.529	15.054
Skewness	0.121	−0.934	−0.089	−0.476	−0.047
Kurtosis	−0.129	0.617	−0.959	−0.496	−0.321

*Note.* ^*∗∗*^ *P* < 0.05.

**Table 3:** The results of multiple linear regression analysis on the influencing factors of task load in ICU nurses (*N* = 253).

Variable	*β*	SE	*β′*	*t*-value	*P* value	95% CI (*β*)
Constant	95.750	8.940	—	10.710	<0.001	78.141, 113.359
Self-efficacy	−0.633	0.165	−0.202	−3.831	<0.001	−0.958, −0.307
Intrinsic cognitive load	0.131	0.113	0.065	1.165	0.245	−0.091, 0.354
Extraneous cognitive load	0.922	0.102	0.520	9.048	<0.001	0.721, 1.123

*Note. β* = nonstandardized coefficient; *β′* = standardized coefficient; *R*^2^ = 0.453; adjusted *R*^2^ = 0.438; *F* = 29.027; *P* < 0.001; Durbin–Watson = 1.859.

**Table 4:** Effect analysis of extraneous cognitive load as a mediator between self-efficacy and task load in ICU nurses (*N* = 253).

Effect	Path	*β*	Bias-corrected 95% CI	SE	*t*-value	*P* value
Direct effect	Self-efficacy ⟶ task load	−0.662 (c*′*)	−0.984, −0.341	0.163	−4.057	<0.001

Indirect effect	Self-efficacy ⟶ extraneous cognitive load	−0.728 (a)	−0.929, −0.527	0.102	−7.144	<0.001
	Extraneous cognitive load ⟶ task load	0.971 (b)	0.788, 1.154	0.093	10.467	<0.001

Total effect	Self-efficacy ⟶ task load	−1.369	−1.721, −1.018	0.178	−7.681	<0.001

*Note.* Adjusting for covariates, including age, gender, educational level, and years of ICU working experience.

## Data Availability

The original data used to support the findings of this study are included within the S1 File.

## Supplementary Materials

Supplementary Materials S1 File: the dataset for data analysis.
