# Supplementary material for: Mediator Effects of Cognitive Load on Association between Self-Efficacy and Task Load in Intensive Care Unit Nurses
Source: J Nurs Manag. 2024 Feb 2;2024:5562751. doi: 10.1155/2024/5562751 (PMC11918911; doi:10.1155/2024/5562751)
Supplement: Supplementary Materials — S1 File: the dataset for data analysis. [file 5562751.f1.pdf]

Number	Age	ICU experience (years)	Professional Title	Gender	Ethnicity	Marital status	Education level	ICU specialty nurse
1	42.00	23	3	2	1	1	3	1
2	35.00	11	3	2	1	2	3	2
3	29.00	9	2	2	1	2	3	2
4	29.00	6	2	2	1	2	3	2
5	39.00	11	3	1	1	1	2	2
6	36.00	10	3	1	1	1	2	2
7	31.00	9	2	1	1	2	3	2
8	38.00	16	3	1	1	1	3	1
9	31.00	9	3	1	1	1	3	1
10	33.00	8	2	1	1	1	3	1
11	36.00	14	3	1	1	1	2	2
12	31.00	12	2	1	1	1	2	2
13	34.00	10	3	2	1	1	3	2
14	23.00	1	1	1	6	2	3	2
15	34.00	7	2	1	1	1	2	1
16	33.00	9	2	1	1	1	3	2
17	32.00	10	2	1	2	1	2	2
18	35.00	14	3	1	1	1	3	2
19	27.00	5	2	1	1	2	3	1
20	37.00	15	3	1	1	1	3	2
21	24.00	2	1	1	2	2	3	2
22	37.00	11	3	2	1	1	3	1
23	37.00	13	3	2	1	1	3	2
24	32.00	7	3	2	1	2	3	2
25	34.00	8	3	2	1	1	3	1
26	39.00	17	3	2	1	1	2	2
27	39.00	17	3	2	1	1	2	2
28	38.00	14	3	2	1	1	2	2
29	38.00	12	3	2	1	1	3	2
30	38.00	16	3	1	1	3	3	2
31	39.00	16	3	2	1	1	3	1
32	38.00	11	3	2	1	1	3	2
33	41.00	19	3	2	1	1	3	2
34	34.00	12	3	2	1	1	2	1
35	41.00	18	3	2	1	1	3	1
36	40.00	14	3	1	1	1	3	1
37	34.00	10	3	2	6	1	2	2
38	26.00	4	1	2	1	2	3	1
39	34.00	12	3	2	1	1	2	2
40	25.00	3	1	2	1	2	2	2
41	32.00	7	3	2	1	1	2	2
42	32.00	6	3	2	1	1	2	1
43	38.00	10	3	2	1	1	3	2
44	36.00	15	3	2	1	1	3	2
45	26.00	4	1	2	1	2	3	1
46	31.00	9	3	2	1	2	3	2
47	31.00	9	2	1	1	1	3	2
48	36.00	12	3	2	1	1	3	1
49	38.00	16	3	2	1	1	3	2
50	32.00	9	2	2	1	1	3	2

51	38.00	14	3	2	1	3	3	1
52	31.00	9	2	2	1	2	3	2
53	27.00	5	2	2	1	2	3	1
54	28.00	5	2	2	1	2	3	2
55	32.00	9	2	2	1	1	3	2
56	41.00	9	3	2	1	1	3	1
57	37.00	15	3	2	1	1	3	1
58	32.00	6	2	2	1	1	2	1
59	37.00	15	3	2	1	1	3	2
60	30.00	8	2	2	1	3	3	2
61	32.00	8	2	2	1	1	3	1
62	32.00	12	2	2	1	2	3	2
63	49.00	20	3	2	1	1	2	2
64	31.00	8	3	1	1	1	3	1
65	32.00	12	2	2	1	1	3	2
66	39.00	13	3	2	1	1	2	1
67	26.00	4	2	2	1	2	3	1
68	21.00	1	1	1	1	2	2	1
69	30.00	8	2	2	1	2	3	1
70	32.00	7	3	2	1	3	3	2
71	29.00	7	2	2	1	2	3	2
72	28.00	4	2	2	1	2	2	1
73	41.00	14	3	2	1	1	3	2
74	30.00	8	2	2	1	2	3	1
75	24.00	2	2	2	1	2	2	1
76	33.00	10	2	2	1	1	3	1
77	37.00	12	3	1	1	1	2	2
78	35.00	13	3	2	1	1	3	2
79	25.00	1	2	2	1	2	3	2
80	32.00	10	3	2	1	1	3	1
81	36.00	16	3	2	1	1	3	2
82	35.00	16	3	1	1	1	3	1
83	32.00	6	2	2	1	1	2	1
84	33.00	7	2	2	1	1	3	2
85	37.00	11	3	1	1	1	3	2
86	28.00	6	2	2	1	2	2	2
87	49.00	16	3	2	1	1	3	2
88	32.00	12	2	2	1	1	3	2
89	36.00	16	2	2	1	1	2	2
90	32.00	14	3	1	1	1	3	1
91	31.00	7	2	2	1	1	2	2
92	31.00	9	3	2	1	1	2	2
93	26.00	4	1	2	1	2	3	1
94	31.00	7	2	2	1	2	3	2
95	32.00	12	2	2	1	1	2	1
96	29.00	6	2	2	1	2	3	1
97	35.00	13	2	2	1	1	2	2
98	33.00	12	2	2	1	1	3	1
99	51.00	12	3	1	1	1	3	1
100	27.00	5	1	2	1	2	2	2
101	35.00	13	3	2	1	2	3	2
102	32.00	7	2	2	1	1	2	2
103	41.00	16	3	2	1	1	2	2
104	27.00	4	2	1	1	2	3	1

105	30.00	8	2	1	1	2	3	2
106	49.00	15	3	1	1	1	3	1
107	35.00	12	3	2	1	1	3	1
108	31.00	9	2	2	1	2	3	2
109	37.00	11	3	1	1	1	3	1
110	41.00	12	3	1	1	1	3	2
111	24.00	2	1	1	1	2	3	2
112	25.00	4	1	1	1	1	2	2
113	32.00	11	2	2	1	1	3	1
114	23.00	1	1	1	6	1	2	1
115	23.00	1	1	1	1	2	3	2
116	41.00	12	3	1	1	1	3	2
117	51.00	13	3	1	1	1	3	1
118	23.00	1	1	1	1	2	3	2
119	31.00	6	2	1	1	1	2	1
120	29.00	7	2	2	1	2	3	1
121	36.00	16	2	2	1	1	3	2
122	23.00	1	1	2	2	2	3	1
123	31.00	7	3	2	1	1	3	1
124	32.00	11	3	2	1	1	3	2
125	38.00	16	3	2	1	1	3	1
126	30.00	7	2	2	1	2	3	1
127	35.00	16	3	2	1	1	3	2
128	50.00	18	3	2	1	1	2	2
129	30.00	7	2	2	1	2	3	2
130	51.00	11	3	1	1	1	3	2
131	38.00	16	3	2	1	1	3	2
132	32.00	12	2	1	1	2	3	2
133	25.00	3	1	1	1	2	3	2
134	33.00	9	3	1	1	1	3	1
135	30.00	9	2	1	1	2	2	1
136	40.00	11	3	2	1	1	3	2
137	35.00	9	3	2	1	1	3	1
138	35.00	12	3	1	2	1	3	1
139	34.00	10	2	2	1	1	2	2
140	30.00	8	2	2	1	2	3	1
141	33.00	11	2	2	1	1	3	1
142	31.00	9	2	1	1	1	2	2
143	31.00	6	2	1	1	2	3	1
144	32.00	12	2	1	1	1	2	2
145	23.00	1	1	2	2	2	3	1
146	30.00	7	2	1	1	2	2	2
147	23.00	1	1	2	1	2	2	1
148	23.00	1	1	2	1	2	2	1
149	24.00	2	1	2	1	2	2	2
150	24.00	2	1	2	1	2	3	2
151	24.00	2	1	2	6	2	3	2
152	24.00	2	1	2	1	2	3	1
153	25.00	3	1	2	1	2	3	2
154	24.00	2	2	2	1	2	3	1
155	31.00	10	2	2	1	1	3	1
156	32.00	12	2	2	1	1	2	2
157	33.00	14	2	2	2	1	3	1
158	32.00	6	2	2	1	1	2	2

159	32.00	7	2	2	1	1	3	2
160	32.00	8	2	2	1	1	2	2
161	32.00	9	2	2	1	1	3	2
162	36.00	14	3	1	1	1	2	2
163	40.00	18	3	1	1	1	3	2
164	32.00	12	3	1	1	1	2	1
165	33.00	6	3	1	1	1	3	2
166	25.00	3	1	1	1	2	2	2
167	26.00	4	1	1	1	2	3	1
168	21.00	3	1	2	1	2	2	1
169	28.00	6	2	1	1	2	3	2
170	30.00	12	2	1	1	2	3	2
171	30.00	7	2	1	1	2	3	1
172	30.00	7	2	1	1	2	3	1
173	30.00	7	1	1	2	1	2	2
174	32.00	11	3	2	6	1	3	1
175	34.00	10	2	1	1	1	2	2
176	31.00	9	3	2	1	2	3	2
177	21.00	1	3	2	1	1	3	1
178	38.00	15	3	1	1	1	2	1
179	33.00	10	2	1	6	1	2	1
180	35.00	12	3	1	1	3	3	2
181	39.00	15	3	1	1	1	3	2
182	42.00	23	2	2	1	1	3	2
183	52.00	29	3	2	1	1	2	2
184	46.00	25	3	2	1	1	3	1
185	39.00	17	3	2	1	1	3	1
186	33.00	10	2	2	1	1	3	2
187	38.00	16	3	1	1	1	3	1
188	25.00	3	1	1	1	2	3	1
189	21.00	1	2	2	1	2	3	2
190	33.00	11	2	2	1	2	2	2
191	23.00	10	1	1	1	2	2	2
192	32.00	9	2	2	1	1	3	1
193	27.00	2	1	1	1	1	2	2
194	31.00	7	2	2	1	1	3	2
195	27.00	4	2	1	1	2	3	1
196	39.00	17	3	1	1	1	3	2
197	33.00	11	3	2	1	1	3	2
198	31.00	9	3	2	1	2	3	2
199	31.00	10	2	2	1	2	3	2
200	37.00	16	3	1	1	1	3	2
201	31.00	9	2	2	1	1	3	2
202	34.00	10	3	2	1	1	2	2
203	33.00	8	2	2	1	2	3	2
204	47.00	28	3	2	1	1	3	1
205	49.00	29	3	2	1	1	2	2
206	35.00	12	3	2	1	1	3	1
207	26.00	3	1	1	1	2	2	2
208	26.00	4	2	1	1	2	2	1
209	28.00	3	2	1	1	1	3	2
210	32.00	12	3	2	1	1	3	1
211	35.00	13	3	1	1	3	3	2
212	31.00	7	2	2	1	1	3	2

213	33.00	11	2	2	1	2	3	1
214	21.00	1	1	1	1	2	2	1
215	29.00	9	2	2	1	2	3	2
216	32.00	10	2	2	1	1	2	2
217	32.00	9	2	2	1	1	3	1
218	28.00	5	1	2	1	1	2	2
219	32.00	8	2	2	1	1	3	2
220	26.00	4	1	1	1	1	2	2
221	25.00	4	3	2	1	1	3	2
222	34.00	13	3	2	1	1	3	1
223	32.00	10	3	2	1	1	3	1
224	26.00	2	2	2	1	2	3	2
225	31.00	8	2	2	1	1	3	1
226	38.00	16	3	1	1	1	3	1
227	34.00	10	2	1	1	1	2	1
228	36.00	16	3	1	1	1	3	1
229	38.00	16	3	1	1	3	3	2
230	32.00	12	2	1	1	1	3	1
231	51.00	16	3	2	1	1	3	1
232	33.00	14	2	2	1	1	3	2
233	40.00	12	3	2	1	1	3	1
234	41.00	16	3	2	1	1	3	2
235	25.00	3	1	2	1	2	2	2
236	33.00	13	3	2	6	1	3	2
237	31.00	12	2	2	1	1	2	2
238	51.00	14	3	2	1	1	2	2
239	33.00	10	3	2	1	1	3	1
240	33.00	11	3	2	1	1	3	2
241	37.00	11	3	2	1	1	3	2
242	32.00	9	3	2	1	1	2	1
243	28.00	6	2	2	1	2	3	2
244	26.00	4	1	2	1	2	3	1
245	32.00	7	3	2	1	1	2	2
246	30.00	11	2	2	1	2	3	1
247	51.00	16	3	1	1	1	3	2
248	30.00	8	2	1	1	2	3	1
249	27.00	5	1	1	1	2	3	2
250	40.00	15	3	1	1	1	3	1
251	37.00	16	3	1	1	1	2	2
252	30.00	12	2	2	1	2	3	2
253	31.00	9	2	2	1	1	3	2

Living situation	Average overtime hours per week	Working night shifts	Number of night shifts per month	Number of patients cared for during the day shift	Number of patients cared for during the night shift	SE1	SE2	SE3
1	5	2	0	2	0	2	2	2
1	2	1	7	2	2	3	1	3
3	4	1	8	2	2	2	2	1
3	4	1	8	2	2	2	2	1
1	2	1	0	2	2	2	2	1
1	2	1	0	2	2	2	2	1
1	3	1	8	2	2	2	2	1
1	2	1	0	2	2	2	2	1
1	4	1	8	2	2	2	2	2
1	4	1	8	2	2	2	2	2
1	3	1	8	2	2	2	2	2
1	1	1	8	2	2	2	2	2
1	2	1	7	2	2	3	2	3
2	2	1	7	2	2	2	2	2
1	4	1	8	2	2	2	2	2
1	4	1	8	2	2	2	2	2
1	4	1	8	2	2	2	2	2
1	4	1	8	2	2	2	2	2
3	2	1	9	2	2	2	2	2
1	2	2	0	2	2	2	2	3
1	2	1	7	2	2	2	2	3
1	3	1	8	2	2	2	3	1
1	3	1	8	2	2	2	3	1
1	3	1	8	2	2	2	2	2
1	3	1	8	2	2	2	3	2
1	3	1	8	2	2	2	3	2
1	3	1	8	2	2	2	3	2
1	3	1	8	2	2	2	3	2
1	3	1	8	2	2	2	3	2
2	4	1	8	4	3	2	3	2
1	3	1	8	2	2	2	3	2
1	3	1	8	2	2	2	3	2
1	2	2	6	2	2	2	3	2
1	2	1	0	2	2	2	3	3
1	4	2	0	2	0	3	1	2
1	2	1	0	3	3	2	3	3
1	3	1	8	2	2	2	4	1
1	1	1	10	2	2	2	4	2
1	1	1	10	2	2	2	4	2
1	1	1	10	2	2	2	4	2
1	3	1	8	2	2	2	4	2
1	1	1	9	2	2	2	4	3
1	3	1	8	2	2	3	1	2
1	3	1	8	2	2	3	1	3
1	1	1	10	2	2	3	1	3
1	4	1	8	2	2	4	2	3
1	3	1	8	2	2	3	2	1
1	3	1	8	2	2	3	2	1
1	3	1	8	2	2	3	2	1
1	4	1	8	2	2	3	2	1

2	4	1	8	2	2	3	2	2
1	3	1	8	2	2	3	2	2
3	4	1	8	2	2	3	2	2
3	4	1	8	2	2	3	2	2
1	4	1	8	2	2	3	2	3
1	2	2	11	2	2	3	2	3
1	3	1	9	2	2	2	2	1
1	4	1	8	2	2	3	2	3
1	5	1	11	2	2	3	2	4
2	2	1	9	2	2	3	3	1
1	4	1	8	2	2	3	3	1
2	2	1	9	2	2	3	3	1
1	2	2	2	2	2	3	3	2
1	4	1	8	3	2	3	3	2
1	4	1	8	2	2	3	3	2
1	3	2	2	2	2	3	3	2
3	2	1	9	2	2	3	3	2
3	2	1	6	1	1	3	3	3
3	2	1	9	2	2	3	3	2
1	1	1	9	2	2	3	3	2
3	4	1	8	2	2	3	3	2
3	4	1	8	2	2	3	3	2
1	2	2	0	2	2	3	3	2
2	2	1	9	2	2	3	3	3
1	2	1	7	2	2	3	3	3
1	4	1	8	2	2	3	3	3
1	5	1	8	4	2	3	3	3
1	5	1	11	2	2	3	3	3
3	3	1	8	2	2	3	3	3
1	1	1	11	2	2	3	3	3
1	4	1	8	3	3	3	3	3
1	5	1	8	3	2	3	3	3
1	4	1	8	2	2	3	3	3
1	4	1	8	2	2	3	3	3
1	5	1	8	3	2	3	3	4
3	4	1	8	2	2	3	4	1
1	2	2	6	2	2	3	4	2
1	4	1	8	2	2	3	4	2
1	4	1	8	2	2	3	4	2
1	4	1	8	2	2	3	2	3
1	1	1	8	2	2	3	4	3
1	4	1	8	3	2	3	4	3
1	1	1	10	2	2	3	4	3
1	1	1	8	2	2	3	4	3
1	4	1	8	2	2	3	4	3
3	4	1	8	2	2	3	4	3
1	4	1	8	2	2	3	4	3
1	4	1	8	2	2	3	4	3
1	2	2	2	2	2	4	1	1
1	5	1	8	2	2	4	1	1
3	2	1	10	2	2	3	2	2
1	5	1	8	2	2	4	1	1
1	2	2	0	2	2	4	1	1
3	2	1	9	2	2	4	1	1

3	2	1	9	2	2	4	1	1
1	3	2	0	2	2	4	1	2
1	5	1	8	2	2	4	1	2
1	5	1	8	2	2	4	1	2
1	2	2	0	2	2	4	1	2
1	2	2	0	2	2	4	1	2
1	2	1	7	2	2	4	1	2
1	2	1	8	2	2	3	3	3
1	1	2	8	2	2	3	1	1
1	2	1	7	2	2	4	1	2
1	2	1	6	2	2	4	1	2
1	2	2	0	2	2	4	1	2
1	2	2	2	2	2	4	1	3
1	2	1	6	2	2	4	1	3
1	3	1	8	2	2	4	2	2
3	4	1	8	2	2	4	2	2
1	4	1	8	2	2	4	2	4
1	2	1	6	2	2	4	3	1
1	4	1	8	4	3	4	3	2
1	4	1	12	2	2	2	2	2
1	2	1	6	2	2	4	3	2
3	4	1	8	2	2	4	3	2
1	2	1	0	2	2	4	3	2
1	3	2	0	2	2	4	3	2
3	4	1	8	2	2	4	3	2
1	3	2	0	2	2	4	3	3
1	2	1	6	2	2	4	3	3
2	2	1	9	2	2	4	3	3
3	2	1	9	2	2	4	3	3
1	3	1	11	2	2	4	3	3
2	3	1	8	2	2	3	1	1
1	3	2	6	2	2	4	3	3
1	5	1	8	2	2	4	3	3
1	5	1	8	3	2	4	3	3
1	4	1	8	2	2	4	3	4
1	2	1	7	2	2	4	4	2
1	4	1	8	2	2	4	4	3
1	5	1	8	2	2	3	2	2
1	5	1	8	2	2	2	3	2
1	5	1	8	2	2	3	2	1
1	2	1	7	1	1	3	3	2
2	2	1	8	2	2	4	4	3
1	2	1	7	1	1	1	3	3
1	2	1	7	1	1	4	3	2
1	2	1	7	1	1	3	2	1
1	2	1	7	1	1	4	3	2
1	2	1	7	1	1	3	2	2
1	2	1	7	1	1	4	2	2
1	2	1	7	1	1	2	1	1
1	2	1	7	1	1	3	3	2
1	3	1	8	1	1	1	2	4
1	3	1	8	1	1	3	1	1
1	1	1	8	2	2	4	3	3
1	3	1	8	1	1	3	3	2

1	3	1	8	1	1	3	2	3
1	3	1	8	1	1	3	2	3
1	3	1	8	1	1	2	1	2
1	3	1	12	1	1	1	2	3
1	3	2	11	1	1	3	3	2
1	1	1	9	2	1	2	3	1
1	1	1	10	2	1	2	3	2
1	1	1	10	2	1	2	1	3
1	1	1	10	2	1	3	4	2
3	4	1	6	2	2	3	1	1
3	4	1	8	2	1	2	2	1
3	4	1	8	2	1	2	4	3
3	4	1	8	2	1	2	3	3
3	4	1	8	2	1	2	2	2
1	4	1	8	2	2	3	2	2
1	2	1	8	2	2	4	2	3
1	3	1	8	2	2	3	1	1
1	4	1	8	2	2	3	2	2
1	4	1	6	2	2	3	2	2
1	2	1	7	2	2	2	2	2
1	4	1	10	2	2	3	2	2
1	3	1	8	2	2	4	3	3
1	3	1	9	3	2	1	1	1
1	2	2	0	2	0	4	1	2
1	2	2	0	2	0	4	1	2
1	2	2	0	2	0	4	1	2
1	4	1	8	2	2	3	2	1
1	5	1	12	2	2	3	3	3
1	1	1	8	2	0	3	2	1
1	4	1	6	2	2	4	3	2
1	3	1	10	2	2	2	2	2
3	2	1	9	3	3	3	1	1
3	1	1	8	2	2	4	2	3
1	2	1	9	2	2	4	1	1
1	5	1	8	2	2	3	2	2
1	5	1	8	2	2	3	2	2
3	1	1	8	2	2	2	2	2
1	2	1	9	2	2	3	2	2
1	3	1	2	2	2	3	2	2
2	2	1	8	2	2	4	2	3
1	1	1	8	2	2	3	3	2
1	2	1	8	2	2	3	1	1
1	2	1	8	2	2	1	2	4
1	2	1	8	2	2	3	3	2
2	4	1	10	2	2	2	1	1
1	2	2	0	2	0	4	2	2
1	2	2	0	2	0	3	2	1
1	4	2	8	2	2	4	3	2
1	4	1	2	2	2	2	2	2
3	4	1	8	2	2	3	1	3
1	4	1	8	2	2	3	2	2
1	5	1	10	4	2	3	2	2
1	3	1	7	2	2	4	4	2
1	4	1	7	2	2	4	3	3

1	3	1	8	2	2	2	4	2
3	4	1	8	1	1	3	3	3
1	1	1	8	2	2	4	3	2
1	2	1	7	2	2	3	2	1
1	3	2	0	2	0	4	3	1
1	1	1	7	2	2	3	4	3
1	2	1	8	3	2	3	1	1
1	1	1	8	2	2	4	4	4
1	3	1	8	2	2	2	1	1
1	3	1	9	2	2	2	3	1
1	5	1	8	2	2	3	2	1
1	4	1	8	2	2	4	2	1
1	2	1	8	2	2	1	2	3
1	4	1	8	2	2	3	2	2
1	3	1	8	2	2	3	1	1
1	3	1	8	2	2	1	2	1
2	4	1	8	2	2	1	2	1
1	4	1	8	2	2	1	2	1
1	2	2	2	2	2	1	3	2
1	3	1	8	2	2	2	2	2
1	2	2	0	2	2	1	3	2
1	2	2	0	2	2	1	3	3
3	2	1	9	2	2	1	4	1
1	1	1	10	2	2	1	4	1
1	1	1	8	2	2	1	4	1
1	3	2	0	2	2	1	4	1
1	1	1	10	2	2	1	4	1
1	1	1	10	2	2	1	4	1
1	2	2	6	2	2	1	4	1
1	1	1	10	2	2	1	4	1
3	2	1	11	2	2	3	1	2
1	1	1	10	2	2	1	4	2
1	3	1	8	2	2	1	4	2
2	2	1	9	2	2	1	4	3
1	2	1	6	2	2	2	1	2
3	4	1	8	2	2	2	1	3
1	5	1	8	2	2	2	1	4
1	2	2	0	2	2	2	2	1
1	2	1	6	2	2	2	2	1
3	4	1	8	2	2	2	2	1
1	3	1	8	2	2	2	2	1

SE4	SE5	SE6	SE7	SE8	SE9	SE10	ICL1	ICL2
3	3	3	3	3	3	3	10	6
2	2	3	3	2	2	2	8	8
3	1	2	3	2	3	2	11	9
3	2	3	3	3	2	2	8	9
3	2	3	3	4	4	3	9	3
3	3	2	3	3	3	3	11	7
3	3	3	2	2	3	3	6	5
3	3	4	3	2	2	1	11	8
2	1	2	1	2	2	1	11	11
2	2	2	2	2	2	2	6	6
2	2	2	2	2	2	2	8	8
2	2	2	2	2	2	2	9	8
2	2	2	2	2	2	2	11	9
2	2	2	2	2	2	3	5	7
2	3	3	3	2	2	2	7	7
3	2	2	2	2	2	3	11	9
3	3	2	3	3	3	3	10	10
3	3	3	3	3	3	3	8	7
3	3	4	3	4	3	3	5	5
3	2	3	2	2	2	2	8	7
3	3	4	3	2	4	3	7	8
1	3	1	2	2	2	1	7	7
2	3	3	3	3	3	1	6	6
3	3	2	2	2	2	2	11	10
2	1	2	2	2	2	2	9	9
2	2	1	2	1	1	1	11	11
2	2	1	3	3	3	2	9	8
2	2	2	2	2	1	2	6	6
2	2	2	2	2	2	2	10	10
2	2	3	3	2	4	4	9	10
3	2	3	3	3	3	3	6	10
3	3	3	3	3	2	2	10	9
4	3	3	3	3	2	3	9	7
4	3	3	3	3	3	3	8	9
3	3	4	3	3	4	3	5	5
4	4	4	4	3	4	4	1	1
3	3	3	3	2	3	2	9	9
2	2	3	3	2	2	3	7	8
2	2	3	3	3	3	3	11	10
3	2	3	3	3	3	3	7	10
3	3	2	2	2	2	2	8	9
2	2	2	2	2	3	2	2	5
2	4	2	2	4	3	3	11	11
2	2	2	2	2	2	2	9	9
3	3	3	3	3	3	4	7	8
4	4	4	4	4	4	3	8	8
1	3	2	1	2	2	1	6	8
2	2	1	1	2	2	1	11	11
3	2	1	2	1	2	1	9	10
3	2	4	2	3	2	3	2	9

2	3	3	3	2	2	2	11	11
2	3	3	3	3	3	3	1	1
3	3	2	3	2	3	2	5	5
3	3	3	2	2	2	3	10	10
2	2	3	4	4	4	4	2	2
2	3	3	1	2	1	2	7	8
3	3	3	3	2	3	2	6	6
3	2	4	4	4	3	3	6	6
3	3	4	4	3	4	4	11	11
1	1	3	3	1	1	2	11	11
2	2	2	2	2	2	2	10	10
2	3	3	1	2	3	3	7	6
1	2	3	4	2	2	2	11	11
2	2	3	2	1	2	2	8	8
2	2	3	3	3	3	3	8	11
2	3	3	3	2	2	2	11	11
2	3	4	3	2	3	3	8	6
3	3	3	3	3	3	3	8	9
2	3	4	4	2	4	4	9	11
3	1	1	2	2	2	2	5	8
3	3	2	3	3	3	3	8	9
3	3	3	3	3	3	3	8	8
3	3	3	3	3	3	3	8	10
1	2	2	1	2	3	2	10	10
2	2	2	1	2	1	2	6	8
2	2	2	2	2	2	1	6	9
2	3	3	2	2	1	1	7	8
3	3	3	3	3	3	3	9	8
3	3	3	3	3	3	4	10	9
3	3	3	3	3	4	4	11	11
4	3	4	2	3	4	2	6	7
4	4	3	4	4	4	2	7	7
4	4	4	4	3	4	4	1	1
4	4	4	4	4	4	3	5	5
3	3	4	3	4	3	3	7	5
3	2	2	2	3	2	2	3	3
2	2	2	1	2	2	2	11	10
2	2	2	2	2	2	2	9	7
2	2	2	2	2	3	2	11	11
3	3	3	3	3	3	3	2	2
2	3	3	3	3	3	3	8	8
2	4	3	3	3	2	4	5	5
3	2	3	2	3	3	3	1	3
3	3	3	3	2	3	3	9	7
3	3	3	3	3	3	3	6	5
3	3	3	3	3	3	3	6	9
3	3	3	3	3	3	3	8	10
3	4	3	4	4	4	4	6	5
1	2	3	3	1	1	1	11	11
2	2	1	2	2	2	2	6	6
3	2	3	3	3	3	1	8	8
2	2	2	2	2	3	2	6	8
3	2	3	2	3	3	2	6	8
3	3	3	2	3	2	2	8	9

3	3	3	3	3	3	3	10	10
2	2	2	2	2	2	2	7	7
2	2	2	2	2	2	2	9	10
2	2	2	2	3	3	2	7	7
2	3	2	2	2	2	3	6	6
2	4	3	4	3	4	4	9	10
3	2	2	2	2	2	2	8	10
2	2	2	2	2	2	2	3	3
3	3	3	3	2	3	2	6	6
3	2	2	2	3	3	3	6	6
3	2	3	3	3	3	2	9	9
3	3	3	3	3	3	3	8	7
2	2	2	2	2	2	2	9	10
3	3	3	3	3	3	3	2	1
2	2	4	2	2	3	3	11	11
3	2	1	3	3	3	3	7	7
3	3	4	3	4	4	3	11	11
2	2	2	2	3	3	2	6	7
1	3	3	2	3	3	2	9	8
2	2	2	2	2	2	2	8	9
2	1	1	2	2	2	1	7	8
2	2	2	1	2	2	2	11	10
2	2	2	3	3	3	1	10	7
2	3	2	2	3	3	3	9	9
2	3	3	2	3	3	3	8	7
2	1	2	2	2	2	2	5	5
2	2	1	2	1	2	2	10	9
3	2	3	3	3	3	3	8	8
3	2	3	3	3	3	3	8	8
3	3	3	2	2	3	3	9	11
1	1	1	3	3	3	3	11	11
3	3	3	2	3	3	4	9	10
3	3	3	3	3	3	3	9	9
4	3	4	4	4	3	4	6	5
3	3	3	3	3	3	3	11	11
2	3	3	3	3	2	3	8	10
4	3	3	3	3	3	3	6	5
2	2	2	1	2	3	2	5	6
3	3	3	2	3	3	2	10	9
3	3	3	2	2	3	1	10	9
3	2	2	2	3	3	2	6	6
3	3	3	3	3	3	3	11	11
3	3	3	2	3	3	3	1	1
3	2	3	2	2	2	1	3	4
2	1	3	2	1	2	1	11	11
2	3	2	2	2	2	2	7	8
3	3	3	2	3	3	2	8	8
3	4	3	2	3	3	3	8	9
2	3	1	1	2	2	1	11	11
3	2	4	1	1	2	1	10	11
4	3	4	4	3	3	3	1	2
3	3	2	4	3	3	2	10	11
3	3	3	3	3	3	3	6	4
3	2	2	3	4	3	3	7	8

4	3	4	4	4	4	4	1	1
2	2	3	2	2	2	1	8	6
2	2	3	2	3	4	2	7	8
1	2	2	2	2	3	2	8	8
2	2	2	3	2	3	2	11	11
3	4	2	3	3	3	3	9	9
2	2	3	3	3	2	2	10	11
3	3	1	2	3	1	3	4	4
3	1	3	3	2	3	3	10	11
1	2	1	1	1	1	1	11	11
1	1	1	1	1	1	2	11	10
3	3	4	3	3	3	3	5	4
4	4	4	4	4	4	4	9	10
3	4	4	3	3	3	3	11	10
3	3	2	3	3	3	3	11	11
3	3	4	4	3	3	3	7	6
3	1	4	3	4	4	3	9	9
2	2	2	2	2	2	2	10	10
2	3	2	2	2	3	3	7	7
2	2	3	3	2	3	2	8	8
2	3	3	4	4	4	4	10	10
3	3	3	3	3	3	3	9	9
3	3	1	4	3	3	3	5	4
2	3	3	3	2	2	2	11	11
3	3	3	3	2	2	2	8	9
3	3	3	3	2	2	2	5	5
2	2	2	2	2	2	1	7	7
3	3	3	3	3	3	3	8	8
3	3	3	3	3	3	3	10	9
2	2	2	3	2	2	2	11	11
2	2	2	2	2	2	2	9	8
3	2	3	2	3	3	2	8	8
2	2	3	2	2	1	1	9	6
3	3	2	2	3	3	3	9	9
3	2	2	2	2	2	2	9	9
2	2	2	3	3	2	2	10	7
2	2	2	2	2	2	2	6	5
2	2	2	2	2	2	2	7	6
3	3	3	3	3	3	3	8	8
4	3	4	4	4	4	4	1	1
3	2	3	3	3	3	3	8	8
3	3	3	3	3	3	2	11	11
4	3	4	4	3	3	3	2	2
3	2	3	1	2	2	1	11	11
2	1	1	2	2	2	1	11	11
3	3	3	3	3	3	3	9	9
3	3	3	3	2	2	2	8	8
2	2	3	3	2	3	3	10	9
2	2	2	1	1	2	1	11	11
3	3	4	3	3	3	3	7	7
3	2	3	3	3	3	2	9	8
3	3	3	3	3	3	2	9	8
3	3	3	4	4	4	3	11	11
3	3	4	4	4	4	4	6	6

2	2	2	4	2	3	3	11	11
3	3	3	3	3	3	3	9	8
2	2	2	2	2	2	2	8	8
1	1	3	3	1	2	1	11	11
3	2	3	3	2	2	1	4	4
3	2	3	3	2	3	3	9	9
3	1	3	3	2	2	2	10	10
3	3	3	3	3	3	3	6	6
3	1	2	2	2	2	1	9	9
1	3	1	2	2	2	1	6	6
2	2	2	2	2	3	2	8	6
2	2	2	2	2	2	2	9	9
3	3	3	3	3	3	3	1	1
3	2	2	3	3	3	2	7	6
3	3	3	3	2	3	1	11	9
2	1	2	1	1	2	2	11	11
2	3	1	4	3	3	3	3	4
3	1	2	2	2	2	1	10	9
2	3	3	2	2	4	2	11	11
3	3	3	3	3	3	2	11	9
3	3	3	2	2	4	2	10	11
3	2	3	2	4	2	1	4	5
1	1	1	1	2	1	1	11	11
1	1	1	3	2	3	3	11	11
2	2	2	2	2	2	3	5	6
2	3	3	2	1	2	2	9	10
3	1	2	3	3	4	4	8	10
3	3	1	3	3	2	1	10	10
3	3	3	2	3	2	2	4	7
3	3	3	3	2	2	3	11	7
2	2	2	2	2	3	2	6	6
3	2	2	2	2	2	2	10	8
3	2	4	3	3	4	3	8	9
2	2	3	3	3	2	2	8	7
3	3	3	3	3	2	3	11	10
3	3	3	3	3	3	3	1	1
4	3	4	3	3	4	3	1	1
1	1	1	1	2	1	1	11	11
1	1	2	3	2	3	3	11	11
2	3	3	3	3	3	2	7	5
2	3	4	1	3	3	3	4	5

ICL3	ECL1	ECL2	ECL3	GCL1	GCL2	GCL3	GCL4	mentalde mand
6	7	7	7	10	10	10	9	13
7	7	6	6	6	6	6	6	12
10	7	7	6	8	9	7	10	20
7	1	1	3	11	11	11	11	17
9	2	2	2	11	11	11	11	19
11	7	8	8	10	8	11	11	20
7	3	8	5	6	6	6	7	9
9	1	2	1	11	11	11	11	18
11	11	11	11	11	11	11	11	20
5	6	8	9	6	8	7	7	10
10	7	10	8	9	9	9	7	13
9	8	9	9	8	9	9	10	16
10	6	6	6	6	6	6	6	11
6	2	3	6	6	7	6	5	13
10	6	6	6	8	7	7	8	14
10	9	11	7	9	9	8	10	16
6	3	9	7	6	7	6	5	13
7	7	7	7	10	10	9	10	11
5	2	1	1	11	11	11	11	13
8	6	7	6	6	5	6	7	14
6	7	5	8	11	9	9	9	16
4	8	11	5	7	7	9	8	15
6	3	6	7	6	6	7	6	11
9	9	9	9	10	9	8	9	18
8	7	10	9	7	10	9	10	18
11	11	11	11	11	11	11	11	20
7	5	5	8	8	7	8	7	16
5	6	7	6	7	7	7	7	12
10	9	10	8	9	9	9	9	18
10	10	11	10	10	9	10	11	16
6	7	7	7	10	9	10	10	13
7	5	7	7	5	6	6	7	15
7	6	5	7	8	6	10	7	13
7	7	9	8	8	6	10	8	13
5	1	1	2	11	11	11	11	11
1	1	1	1	11	11	11	11	18
8	2	4	3	9	8	9	9	15
4	7	3	6	5	6	7	6	11
10	9	6	7	7	10	10	9	20
9	8	4	10	6	7	8	7	15
7	5	7	6	5	6	7	6	13
2	3	2	4	2	3	4	3	6
11	11	11	10	10	10	8	9	18
8	8	11	6	8	9	9	8	13
6	2	7	11	11	8	5	8	12
8	1	1	2	11	11	11	11	15
4	10	8	6	9	7	8	7	13
11	7	7	9	6	6	7	5	18
9	9	5	5	9	9	8	9	19
9	2	1	3	11	11	9	10	21

11	1	1	1	11	11	11	11	15
1	1	1	1	11	11	10	11	16
5	1	1	1	11	11	11	11	15
10	1	1	1	11	11	11	11	14
2	2	1	2	11	11	11	11	18
9	7	8	9	11	11	11	11	17
6	5	6	5	7	6	6	6	11
7	1	1	1	11	11	11	9	10
11	1	1	4	11	11	10	11	19
11	7	4	4	7	7	6	6	20
7	8	11	8	10	9	9	8	17
7	6	8	7	8	8	8	9	16
11	7	6	6	11	11	11	11	12
11	11	8	8	9	10	9	8	14
10	11	9	11	11	10	9	10	20
11	1	1	1	11	11	11	11	10
9	4	5	4	8	8	7	9	15
8	8	8	8	8	8	8	8	16
10	7	5	8	7	6	6	6	16
8	7	7	6	6	9	5	9	17
8	5	5	6	8	7	9	8	15
11	1	1	1	11	11	11	10	11
9	1	1	1	11	11	11	11	9
10	6	7	5	7	5	6	6	11
8	3	3	3	9	10	8	9	19
9	8	7	9	11	11	10	11	16
9	5	6	7	7	5	6	6	15
10	8	8	11	9	8	11	9	18
9	8	9	8	8	9	9	8	18
11	5	1	6	11	11	11	11	18
6	1	1	1	10	11	10	11	11
10	1	2	1	11	11	11	11	12
1	1	1	1	11	11	11	11	20
5	1	2	1	11	10	11	11	15
6	8	5	5	6	5	7	6	13
6	4	2	3	7	8	9	8	10
10	8	7	7	8	6	10	8	16
8	6	5	7	5	6	7	6	14
11	9	5	5	11	11	11	11	10
2	1	1	1	8	9	9	9	17
9	8	8	8	10	8	6	8	17
4	7	4	6	11	11	11	11	12
2	1	1	1	10	9	7	9	18
8	8	8	8	10	8	8	7	19
3	6	6	5	11	11	11	11	12
9	8	10	6	10	6	8	8	9
9	9	10	8	9	9	9	1	18
5	3	3	2	11	11	9	9	17
11	6	3	6	6	6	8	6	20
9	8	4	2	8	10	8	9	18
8	5	5	5	8	8	8	8	16
8	4	3	2	10	10	7	9	19
10	1	2	2	11	11	11	11	13
7	5	6	7	5	6	7	6	11

6	2	3	4	9	8	9	9	13
7	7	7	6	6	6	8	9	15
11	11	9	10	11	10	10	9	18
9	4	5	4	11	11	10	11	11
8	6	8	7	9	8	8	8	14
11	8	3	9	7	6	6	6	14
9	8	10	9	8	9	10	9	15
3	3	3	3	3	3	3	3	6
6	6	4	4	5	5	5	5	13
7	5	6	5	7	6	5	6	9
8	6	9	7	7	7	7	7	13
8	6	3	4	8	7	8	9	13
11	5	8	5	5	6	6	7	9
3	1	1	1	7	10	9	9	15
11	11	11	10	9	10	9	9	19
10	5	7	3	8	7	9	8	16
11	3	1	2	10	11	11	11	19
10	5	5	3	11	11	10	11	11
3	1	2	3	11	10	10	10	16
9	9	8	9	9	9	9	9	18
6	5	5	4	9	9	8	9	18
10	6	9	7	10	8	8	6	12
7	5	8	2	8	7	9	8	13
7	5	5	6	8	7	7	8	12
8	5	7	6	10	8	6	7	9
5	3	2	4	3	2	3	4	3
11	10	9	11	11	10	9	10	18
8	8	7	8	9	8	7	9	15
9	8	9	7	9	7	8	8	13
7	4	6	7	9	9	8	8	13
11	11	1	11	5	11	11	11	18
9	8	8	9	10	7	9	8	19
10	8	10	7	10	9	7	8	15
5	3	3	2	11	9	9	11	16
11	3	5	4	11	11	11	11	15
9	6	5	5	9	9	8	8	17
7	5	6	7	7	6	6	5	13
7	2	3	6	4	6	8	6	13
7	6	8	5	5	6	7	6	17
9	1	1	2	10	11	11	11	18
7	5	5	6	8	4	6	6	13
11	3	1	8	11	11	11	11	17
1	1	1	1	11	11	10	11	18
5	4	2	3	10	8	7	7	15
11	8	2	5	7	6	7	6	20
9	7	5	6	5	6	7	6	15
10	9	6	7	6	7	7	8	17
10	2	1	1	10	11	11	11	13
11	7	5	11	7	4	7	6	20
10	8	7	7	6	8	9	9	17
3	2	1	2	11	10	11	11	20
8	8	6	9	11	8	11	10	20
4	6	4	7	11	11	11	11	11
8	7	5	6	8	8	6	9	14

1	2	1	1	10	11	11	11	20
10	7	8	9	11	10	11	11	18
9	1	2	2	11	11	11	11	17
10	9	8	8	8	8	10	8	17
11	10	3	6	10	11	11	11	14
8	4	2	3	9	10	9	9	17
12	1	2	1	11	11	11	11	15
6	6	5	4	6	9	8	9	14
10	10	6	6	10	9	8	9	22
11	11	11	11	11	11	11	11	20
11	11	10	11	10	10	11	11	22
5	7	3	7	10	11	10	11	13
11	2	3	2	10	10	11	11	17
11	2	2	2	10	11	11	11	13
9	9	7	6	9	9	9	9	20
6	1	1	1	9	11	11	11	10
3	3	1	2	11	11	11	11	17
10	10	10	10	10	10	10	10	18
6	6	6	9	8	8	9	8	16
8	6	4	8	8	8	7	7	16
10	9	3	8	6	7	6	6	16
9	9	9	9	9	10	9	9	17
5	5	6	4	8	8	8	8	12
11	1	1	1	11	11	11	11	13
8	1	1	1	11	11	11	11	12
5	1	1	1	11	11	11	11	13
7	6	4	4	8	9	9	9	18
8	8	8	8	8	8	8	8	12
7	3	3	3	9	9	8	9	15
11	9	4	6	11	11	11	11	12
9	8	9	8	9	8	9	8	15
8	2	1	2	11	11	11	11	15
9	6	9	9	11	11	11	11	16
2	2	2	2	11	9	11	10	19
9	9	9	9	9	9	9	9	17
6	4	6	3	10	11	11	11	11
6	6	6	7	6	7	7	8	12
8	8	6	6	6	5	9	9	17
7	5	4	4	8	8	8	8	15
1	1	1	1	11	11	11	11	20
7	6	6	6	8	8	8	7	12
7	7	7	9	10	10	10	10	20
2	1	2	2	11	11	11	11	18
9	7	8	7	8	8	8	8	15
11	6	6	11	6	6	6	6	20
9	1	1	1	11	11	11	11	11
8	6	6	6	6	6	6	6	13
10	11	10	10	10	10	10	10	19
11	11	11	11	11	11	11	11	20
7	7	7	6	8	8	8	8	14
9	8	7	7	6	8	7	7	15
8	4	6	6	8	6	8	8	15
11	2	2	2	11	11	10	11	19
4	6	1	1	11	9	11	9	16

11	10	11	11	10	9	9	9	18
7	8	7	9	8	8	8	9	18
8	6	6	6	6	6	6	6	13
11	7	3	5	8	6	6	6	20
4	3	3	3	8	8	8	8	13
9	5	6	5	8	9	9	8	16
10	6	6	8	7	9	8	10	18
6	6	6	6	6	6	6	6	13
10	7	6	6	9	8	9	9	17
6	8	8	8	8	7	8	8	15
8	4	2	3	10	10	7	9	19
9	9	9	9	9	9	9	9	17
1	1	1	1	10	11	11	11	16
6	4	6	6	6	6	6	6	11
8	1	1	2	11	11	11	11	16
11	7	8	8	6	5	6	7	20
7	4	5	6	6	8	9	9	14
9	6	6	7	8	9	9	9	19
11	1	1	1	11	11	11	11	11
6	5	9	5	6	6	6	6	15
9	1	1	1	11	11	11	11	10
3	2	2	5	8	6	10	8	14
11	11	11	11	11	11	11	11	20
11	11	6	6	11	11	5	11	16
7	3	2	6	8	6	4	6	9
11	6	7	7	7	9	8	10	16
3	2	2	2	11	11	11	11	15
8	2	1	1	11	11	11	11	14
7	5	4	5	5	6	5	4	11
11	7	9	7	10	11	9	10	18
6	1	4	6	6	6	6	6	11
9	7	11	9	11	9	7	9	15
8	1	2	1	11	11	11	11	9
8	7	7	5	8	6	4	6	10
10	9	8	5	9	8	10	9	18
1	1	1	1	11	11	10	11	14
4	2	1	2	11	11	11	11	16
11	11	11	11	11	11	11	11	20
11	7	5	11	6	11	10	11	20
6	5	5	4	5	6	5	4	15
5	5	5	5	5	8	8	8	14

physicaldemand	temporaldemand	effort	performance	frustration
15	16	17	19	5
15	14	14	14	14
20	9	19	13	16
20	13	17	1	2
20	16	20	2	13
20	13	19	7	11
20	16	16	17	9
18	16	20	3	0
20	20	20	20	20
15	12	12	14	11
17	15	16	17	14
19	15	18	16	14
11	11	11	11	11
12	10	14	12	10
20	20	20	17	14
19	17	17	18	15
20	20	20	17	11
15	18	15	19	7
13	10	10	2	3
15	12	16	14	12
20	12	18	14	4
20	17	19	17	19
18	14	13	17	11
17	17	17	16	17
18	12	17	15	16
20	20	20	20	20
18	20	18	19	14
11	14	10	14	13
15	19	15	18	17
19	19	18	1	8
13	18	16	20	5
18	19	18	17	11
13	15	15	9	10
9	12	14	9	12
13	12	12	2	1
20	15	20	1	4
16	11	16	16	15
12	8	12	11	10
20	15	14	14	14
20	15	17	12	13
20	20	19	12	13
12	10	14	10	11
20	11	10	13	13
15	17	14	15	18
20	16	14	14	8
15	11	15	1	1
20	20	19	15	19
20	20	19	19	20
9	7	19	7	15
14	14	19	1	5

16	11	19	1	2
18	18	15	15	2
15	10	18	1	3
15	10	19	1	2
16	16	13	20	5
17	15	17	13	16
20	15	15	15	11
14	17	14	7	7
19	19	20	6	9
20	18	20	15	12
15	19	15	19	17
17	13	14	14	15
19	20	12	4	17
18	17	17	11	15
15	19	19	16	19
16	12	20	1	12
19	15	17	12	13
16	15	14	15	15
20	15	15	8	8
20	17	15	11	13
17	20	18	17	11
18	20	18	17	13
20	20	20	1	14
12	8	11	11	11
20	9	12	14	13
18	17	14	18	16
19	14	14	15	16
15	17	19	4	16
17	17	16	16	17
14	20	21	4	4
13	15	17	2	7
16	11	15	13	13
18	17	18	3	1
13	13	13	3	1
13	12	14	13	13
15	14	15	14	20
11	16	17	14	16
16	14	15	12	16
20	20	12	15	18
18	17	18	2	2
13	15	15	12	15
15	15	16	13	12
15	17	19	-1	2
15	16	17	14	17
15	15	16	13	12
13	12	13	9	13
15	17	19	4	16
12	15	13	4	1
20	20	20	13	15
18	18	15	16	16
16	16	16	6	15
19	12	12	14	13
20	17	13	1	6
20	20	20	17	11

17	14	15	17	13
20	20	16	13	11
16	20	16	20	17
18	13	18	13	15
18	16	14	16	13
20	19	17	8	6
20	19	19	20	16
11	14	12	11	11
13	13	13	13	13
13	11	11	13	9
20	19	18	13	11
20	16	17	14	13
13	11	11	13	9
20	17	18	4	0
19	3	19	13	13
14	18	14	8	15
19	20	20	3	11
20	9	18	13	15
15	16	17	1	4
17	15	18	14	16
20	14	16	15	16
15	16	16	13	17
17	16	14	3	4
20	19	17	3	6
17	15	14	15	19
12	14	12	1	10
19	15	19	16	18
19	16	16	17	20
17	15	14	2	5
18	14	15	17	9
18	17	17	17	16
14	17	17	13	17
18	17	16	14	19
15	14	11	7	1
17	20	20	7	4
17	14	15	14	9
13	13	13	12	14
10	11	11	14	12
18	19	19	17	11
16	16	20	3	2
9	12	10	13	11
17	20	20	7	4
18	15	15	15	2
12	15	12	9	11
19	20	19	17	12
17	14	12	17	16
17	17	17	13	13
18	19	19	15	13
18	20	19	19	20
12	15	15	19	16
16	15	12	20	5
18	15	19	5	13
18	15	15	16	12
14	17	10	14	10

18	17	16	3	5
18	15	14	18	16
18	17	11	3	4
13	13	16	17	16
18	18	12	4	6
13	14	13	17	15
14	15	16	3	1
18	15	8	17	8
18	14	14	17	14
20	20	20	20	20
18	19	18	22	20
16	16	12	18	12
13	10	14	3	1
11	11	11	4	1
20	15	15	15	14
16	15	16	5	7
20	18	18	2	15
18	18	18	18	17
16	16	14	14	15
20	20	20	15	14
19	18	17	6	8
18	17	18	7	16
20	13	12	15	8
16	13	20	1	1
15	12	20	1	1
15	12	20	1	1
20	16	17	14	16
12	12	13	12	12
15	14	15	15	15
20	20	12	2	6
15	15	16	15	16
20	15	15	1	4
20	15	16	16	16
14	16	17	1	7
20	18	18	18	18
20	11	20	11	15
13	12	12	12	13
19	20	15	11	13
18	16	17	12	15
20	15	20	1	1
16	15	14	12	10
20	15	19	3	13
18	14	15	18	5
14	16	16	17	16
20	20	20	16	20
20	20	20	13	13
20	20	20	13	13
18	19	18	19	19
20	20	20	20	20
20	14	16	14	6
19	18	18	11	13
19	19	19	15	11
19	19	20	3	12
15	15	12	7	1

20	2	20	13	13
18	16	16	17	17
19	14	14	15	16
20	20	20	15	12
14	14	15	7	11
18	14	15	14	9
20	11	17	13	18
13	13	13	13	13
9	9	17	7	17
20	20	19	17	18
19	12	12	14	13
17	17	17	17	17
20	16	17	13	2
11	11	11	11	11
18	18	20	1	2
20	20	20	17	19
18	15	10	12	11
9	7	19	7	15
16	15	18	1	3
20	20	20	15	11
15	14	18	1	3
11	14	16	4	11
20	20	20	20	20
18	19	15	17	18
12	14	10	12	14
20	13	15	13	20
20	20	16	2	17
18	20	18	1	4
13	15	11	13	15
20	17	17	3	15
12	12	12	12	12
20	20	16	18	20
13	14	10	2	3
15	16	12	14	16
20	15	17	17	12
20	18	15	13	4
18	16	13	18	7
20	20	20	20	20
18	15	19	17	14
13	11	15	13	11
20	11	14	15	6
